# Supplementary figures and images for: Susceptibility rhythm to bacterial endotoxin in myeloid clock-knockout mice
Source: eLife. 2021 Oct 18;10:e62469. doi: 10.7554/eLife.62469 (PMC8598165; doi:10.7554/eLife.62469)

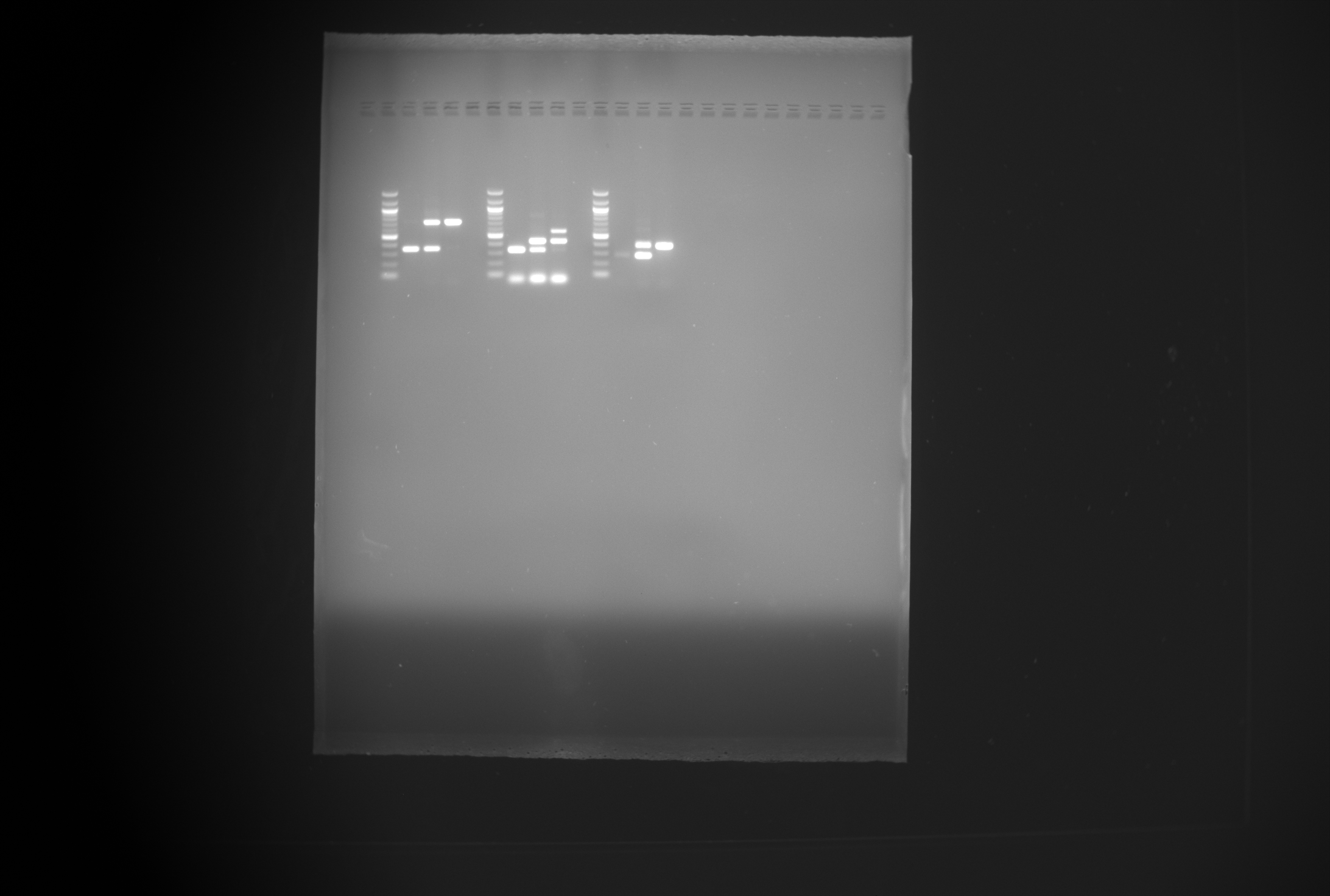

Supplement: Figure 2—figure supplement 1—source data 1. [file elife-62469-fig2-figsupp1-data1.zip › Genotyping gels/LysMcre_Bmalflox_Clockflox_3,1-2._Scan,1-1._Scan.tif]

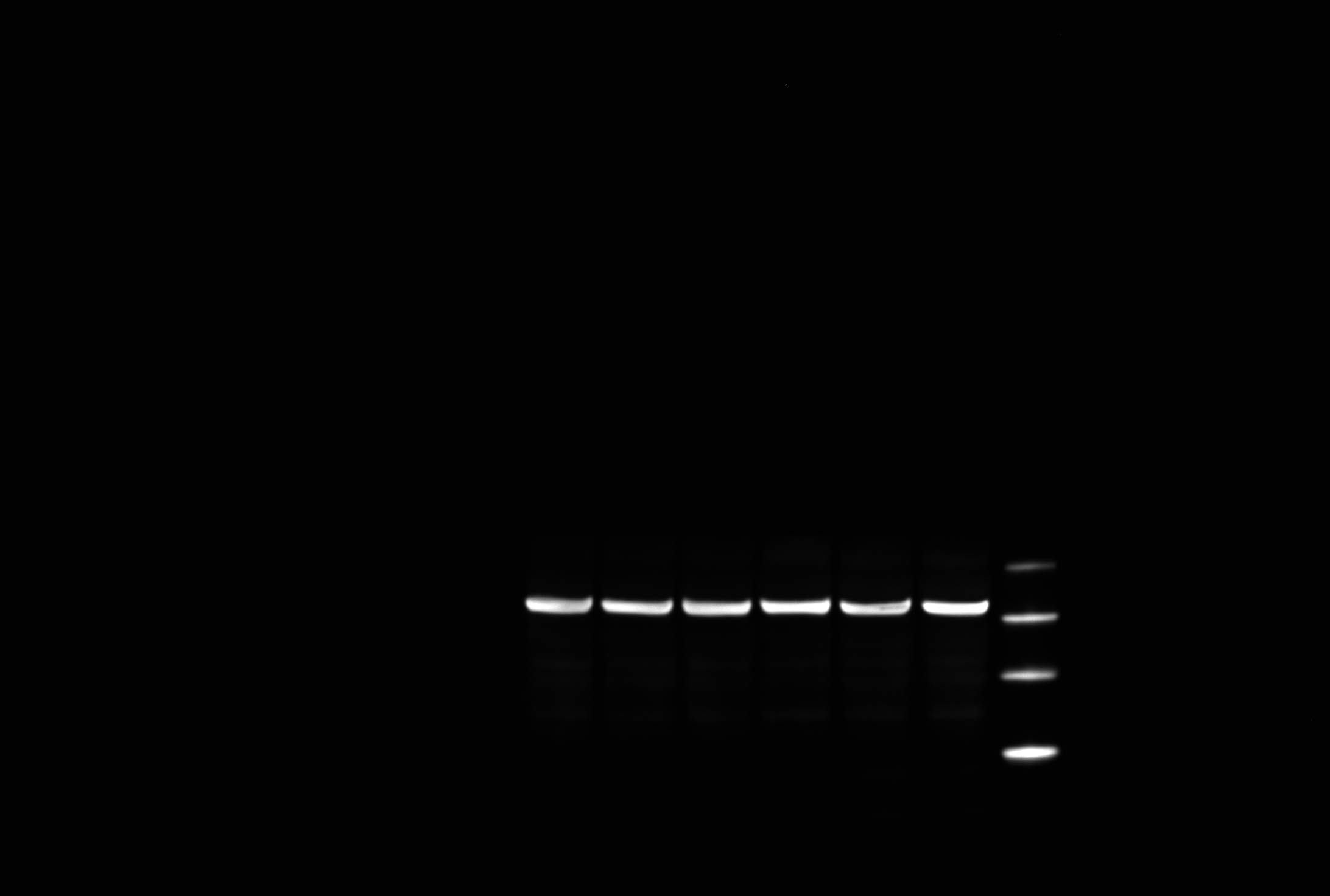

Supplement: Figure 3—source data 1. [file elife-62469-fig3-data1.zip › Western blot data/WB_Fig_3B/liver/Actin Liver/actin 10s,1-1. Scan.tif]

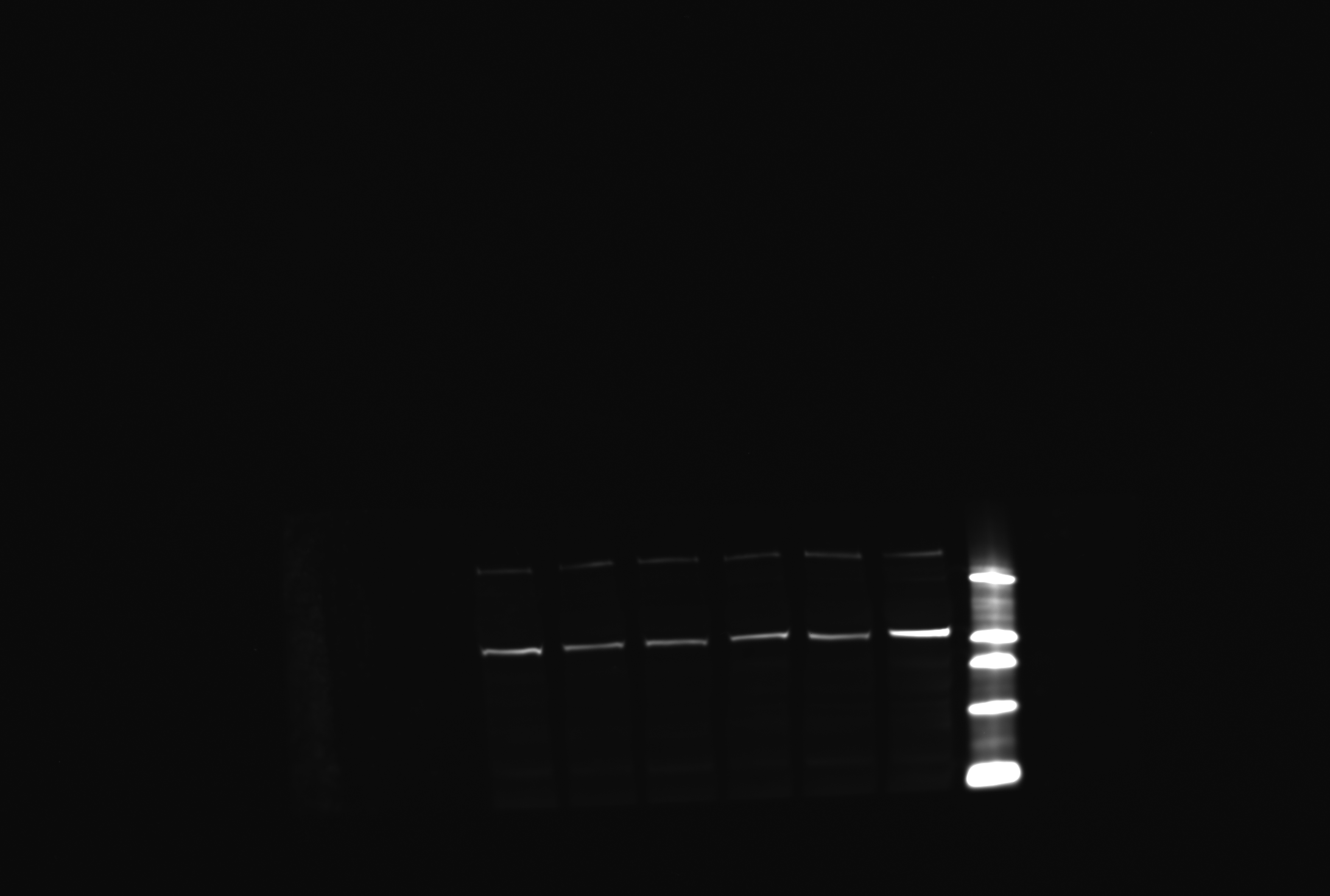

Supplement: Figure 3—source data 1. [file elife-62469-fig3-data1.zip › Western blot data/WB_Fig_3B/liver/Clock Liver/clock,10-10. scan.tif]

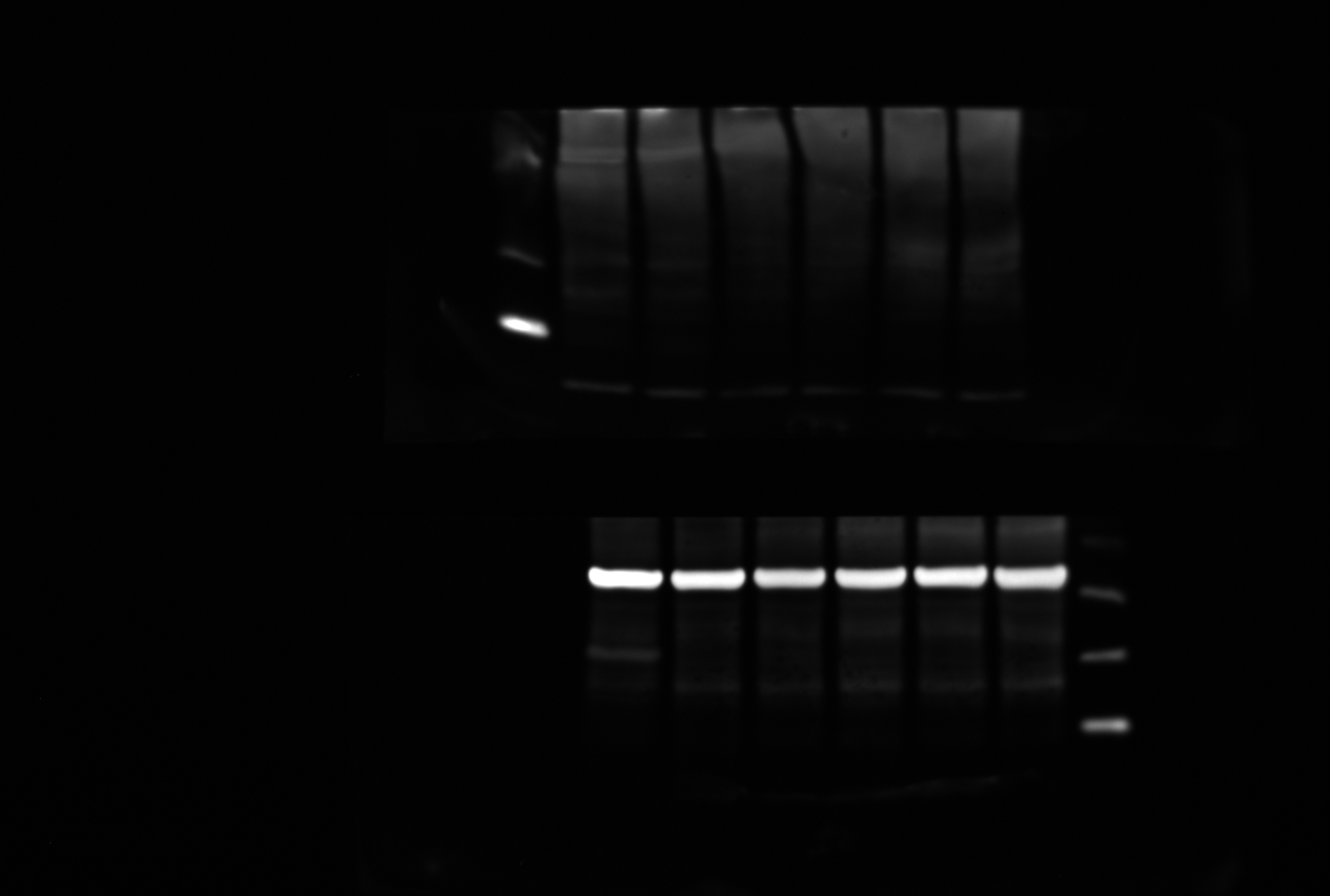

Supplement: Figure 3—source data 1. [file elife-62469-fig3-data1.zip › Western blot data/WB_Fig_3B/peritoneal exsudate cells/Actin PEC/ßactin,4-4. scan.tif]

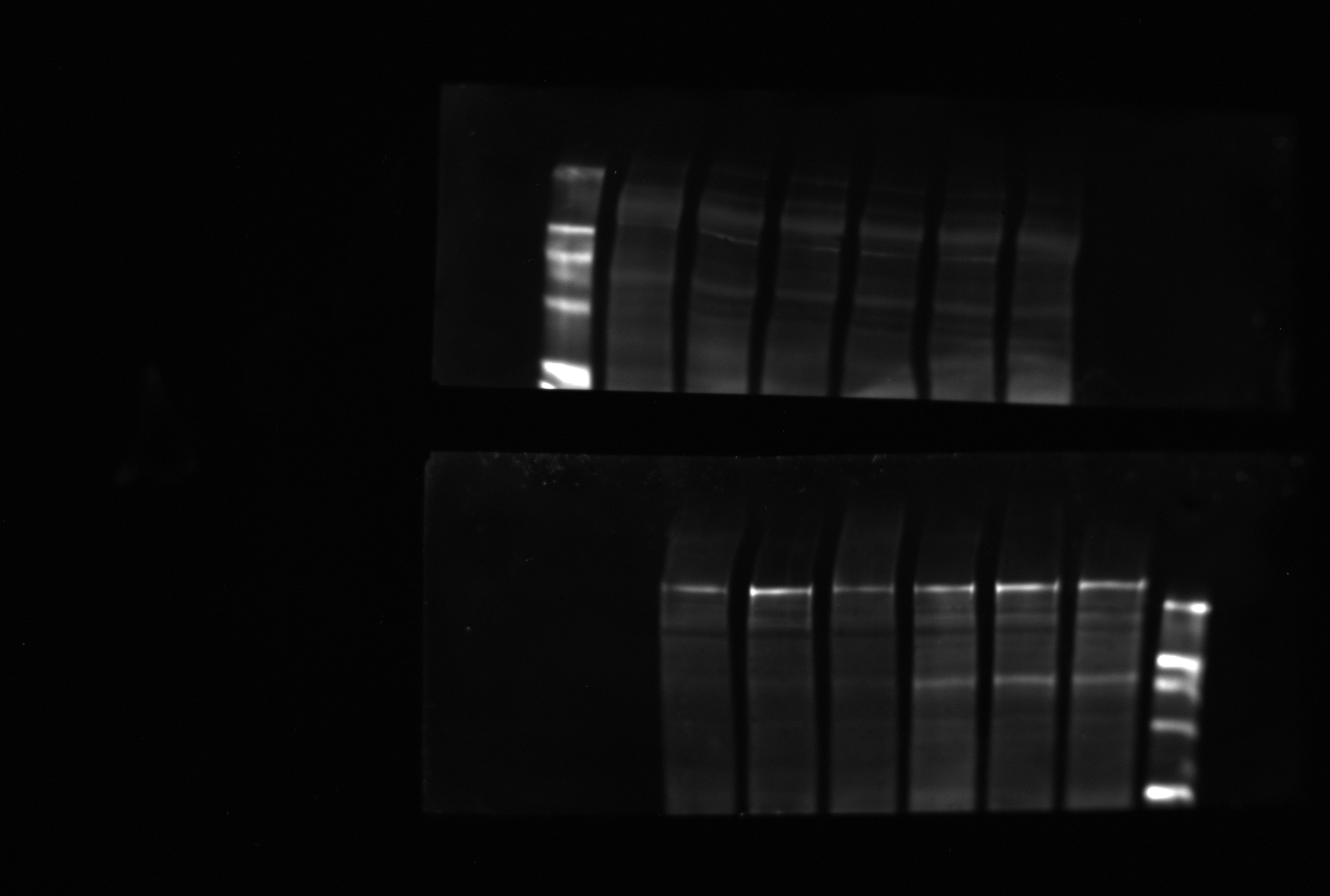

Supplement: Figure 3—source data 1. [file elife-62469-fig3-data1.zip › Western blot data/WB_Fig_3B/peritoneal exsudate cells/Clock PEC/clock,9-9. scan.tif]
